# MetaBar - a tool for consistent contextual data acquisition and standards compliant submission

**DOI:** 10.1186/1471-2105-11-358

**Published:** 2010-06-30

**Authors:** Wolfgang Hankeln, Pier Luigi Buttigieg, Dennis Fink, Renzo Kottmann, Pelin Yilmaz, Frank Oliver Glöckner

**Affiliations:** 1 Microbial Genomics Group, Max Planck Institute for Marine Microbiology, Celsiusstrasse 1, D-28359 Bremen, Germany; 2 Jacobs University Bremen gGmbH, D-28759 Bremen, Germany; 3 Symbiosis Group, Max Planck Institute for Marine Microbiology, Celsiusstrasse 1, D-28359 Bremen, Germany

## Abstract

**Background:**

Environmental sequence datasets are increasing at an exponential rate; however, the vast majority of them lack appropriate descriptors like sampling location, time and depth/altitude: generally referred to as metadata or contextual data. The consistent capture and structured submission of these data is crucial for integrated data analysis and ecosystems modeling. The application MetaBar has been developed, to support consistent contextual data acquisition.

**Results:**

MetaBar is a spreadsheet and web-based software tool designed to assist users in the consistent acquisition, electronic storage, and submission of contextual data associated to their samples. A preconfigured Microsoft^® ^Excel^® ^spreadsheet is used to initiate structured contextual data storage in the field or laboratory. Each sample is given a unique identifier and at any stage the sheets can be uploaded to the MetaBar database server. To label samples, identifiers can be printed as barcodes. An intuitive web interface provides quick access to the contextual data in the MetaBar database as well as user and project management capabilities. Export functions facilitate contextual and sequence data submission to the International Nucleotide Sequence Database Collaboration (INSDC), comprising of the DNA DataBase of Japan (DDBJ), the European Molecular Biology Laboratory database (EMBL) and GenBank. MetaBar requests and stores contextual data in compliance to the Genomic Standards Consortium specifications. The MetaBar open source code base for local installation is available under the GNU General Public License version 3 (GNU GPL3).

**Conclusion:**

The MetaBar software supports the typical workflow from data acquisition and field-sampling to contextual data enriched sequence submission to an INSDC database. The integration with the megx.net marine Ecological Genomics database and portal facilitates georeferenced data integration and metadata-based comparisons of sampling sites as well as interactive data visualization. The ample export functionalities and the INSDC submission support enable exchange of data across disciplines and safeguarding contextual data.

## Background

The technological advancement in molecular biology facilitates investigations of biodiversity and functions on a temporal and geospatial scale. Improved sampling and laboratory methods, together with fast and affordable sequencing technologies [1], provide the framework to create a network of data points capable to answer basic ecological questions such as: 'Who is out there?' and 'What are these organisms doing?' To shed light on the complex interplay, adaptation and survival mechanisms of organisms in times of global change, contextual data describing the surrounding environment of sampling locations are of crucial importance [2]. At the very least, the latitude and longitude (x, y), the depth/altitude (z) in relation to sea level, and the sampling date and time (t) must be provided to allow anchoring molecular sequence data to their environmental context. If every sequence entry in the INSDC databases, comprising of DDBJ, EMBL and GenBank, would be thus georeferenced, researchers would have the post factum opportunity to contextualize these sequences with environmental data [3]. The power of contextual data enriched sequence data sets for the environmental and medical field has been recently documented [4-12].

Unfortunately, a survey in the EMBL sequence repository has shown that only a minor set of sequences are accompanied by a relevant amount of contextual data. For example, latitude, longitude (INSDC: lat_lon), and time (INSDC: collection_date), elements of the key contextual data tuple (x,y,z,t), are only reported in 7.3% and 7.2% of all submissions [Guy Cochrane, personal communication, October 2009]. But even if these data are available, correctness is not guaranteed.

The paucity of sequence associated contextual data has been recognized by the primary database providers and biocuration efforts are currently underway for specific subsets. The National Center for Biotechnology Information (NCBI), for example, curates the Reference Sequence (RefSeq) database which aims to provide a comprehensive, non-redundant, well-annotated set of sequences, including genomic DNA, transcripts and proteins http://www.ncbi.nlm.nih.gov/RefSeq/. The European Molecular Biology Laboratory (EMBL) provides the UniProt/Swiss-Prot Knowledgebase which focuses on high quality protein sequence annotations http://www.ebi.ac.uk/uniprot/ [13]. However, the common aim of these efforts is to enhance the quality of the sequence or protein data and annotations rather than to provide more information on the data processing or the environment where the sample or organism has been taken.

To improve the quantity and quality of contextual data describing the environment of a sample is currently addressed by several projects which systematically collect georeferenced sequence data, environmental parameters, and further curated metadata [14]. SILVA [15] or RDP II [16] are examples for specialized databases that offer users curated and quality checked ribosomal RNA sequences that are often enriched with more reliable contextual and taxonomic information than originally annotated by the sequence submitters.

Furthermore, there are projects which curate the contextual data associated to the primary sequence data to facilitate specific analysis purposes. For example the Genomes OnLine Database (GOLD) collects metadata for ongoing and completed genome sequencing projects [17]. The Visualization and Analysis of Microbial Population Structures (VAMPS) project, with its integrated collection of tools for researchers, aims to visualize and analyze data for microbial population structures and distributions. All the contextual data in VAMPS comes from the MICROBIS database management system of the International Census of Marine Microbes (ICoMM: http://icomm.mbl.edu/microbis/). The megx.net portal http://www.megx.net[18] systematically integrates environmental parameters and sequence data of marine microbial genomes and metagenomes using georeferencing as an anchor.

In 2005 the international Genomic Standards Consortium (GSC) introduced checklists to promote standardized contextual data acquisition and storage. So far the Minimum Information about a Genome (Metagenome) Sequence (MIGS/MIMS) has been published [2] and the Minimum Information about an Environmental Sequence (MIENS) is in development http://gensc.org/gc_wiki/index.php/MIENS. For data exchange, the Genomic Contextual Data Markup Language (GCDML) [19] has been developed. A corollary of these ongoing efforts is the need to support field scientists in the consistent capture, storage and submission of both contextual and sequence data. Handlebar, a lightweight Laboratory Information Management System (LIMS) for the management of barcoded samples, in part addresses this issue by supporting the acquisition and processing of contextual data compliant with GSC standards [20]. The Barcode of Life Database (BOLD) initiative, which aims to identify and classify all eukaryotic life on Earth [21], also includes an advanced data acquisition and submission system. Unfortunately the system only supports phylogenetic markers which serve the eukaryotic domain e.g. the cytochrome c oxidase I (COI), which is only present in Eukarya and absent in the other domains of life, and so far exclude Archaea and Bacteria. Furthermore, it does not support the printing of database identifiers as barcodes to label collected samples.

Even though initiatives and tools exist to enhance the quantity and quality of contextual data subsequent to sequence submission, the amount of contextual data in the INSDC databases remains an issue. In summary, the most likely reasons for the persisting scarcity of consistent contextual data are: (1) Contextual data that are recorded in the field are often not stored electronically in structured databases. Consequently contextual data get rapidly unlinked from the sequence data and finally 'forgotten' in the sequence submission process. (2) There is a lack of automatic quality checking mechanisms active before data submission. Unfortunately, the flood of data entering the public databases prevents any manual curation process. (3) The sheer amount of potential contextual data with respect to the different fields of research ranging from textual data to images or even videos would rapidly exceed the capacities of the INSDC databases. Consequently, only a commonly agreed and standardized subset of data can be stored and made available.

Here the user-centric, web-based tool MetaBar is presented. MetaBar offers all the required features for sample identification and barcode labeling allowing robust sample tracking and inventorying. MetaBar is focused on the acquisition of contextual data recorded during sampling in the field 'offline' using spreadsheets. All recorded contextual data can be subsequently uploaded and consistently stored in an underlying database. The web Graphical User Interface (GUI) provides advanced user management and access to data and barcodes. Vitally, the tool captures GSC standards compliant data and it is integrated into a set of tools to facilitate further data usage such as integration, visualization and analysis available from the Marine Ecological Genomics database and portal, megx.net. Finally, MetaBar supports contextual data enriched sequence submission to the INSDC databases. The tool is not restricted to any given research field or domain of life, but can universally be applied to capture the contextual data of any biological sample. It is designed to support the complete workflow from the sampling event up to the sequence submission to an INSDC database.

## Implementation

### Programming languages, tools and frameworks

MetaBar is programmed in the object-oriented, platform-independent programming language, Java 1.5 http://www.java.com/en/. MetaBar is a multiuser web application using Apache Tomcat http://tomcat.apache.org/, the open source Spring framework http://www.springsource.org/about, jasig CAS http://www.jasig.org/cas, which is used as a central authentication service to implement the user management, and Apache POI http://poi.apache.org/ to parse the Microsoft^® ^Excel^® ^spreadsheets used for data input. Any Java objects generated are stored in a PostgreSQL database using the iBATIS persistence framework http://ibatis.apache.org/. The input fields in the Microsoft^® ^Excel^® ^spreadsheet are validated using Visual Basic (VBA) macros. The web interface has been continuously tested during development using Selenium IDE http://seleniumhq.org/projects/ide/ and JUnit http://www.junit.org/. The source code of the MetaBar application is available under the GNU GPL3 http://www.megx.net/metabar/data/metabar-1.0.tar.gz and as additional file 1 to this publication.

### Core software components

The MetaBar application consists of (1) the Microsoft^® ^Excel^® ^acquisition spreadsheet which is used to capture and auto-correct the contextual data, (2) the MetaBar server which generates and receives the acquisition spreadsheets, parses the data and stores them in (3) MegDB, a PostgreSQL database which is the central database of the megx.net portal [18].

### External software components

MetaBar is integrated into a set of external tools directly accessible from the web interface. The interpolation of environmental physical and chemical parameters of the oceans can be initiated via the WOA05 data extractor of the megx.net portal. On the fly visualization of sampling sites on a world map can be performed using the Genes Mapserver http://www.megx.net/gms and in Google Earth^® ^via the KML export function. The data can be exported prior to sequence submission as a structured comment block for submission to INSDC by the Sequin tool http://www.ncbi.nlm.nih.gov/Sequin/index.html. MetaBar also includes a data export to GCDML [19] for report creation and data exchange.

## Results

**Figure 1:**
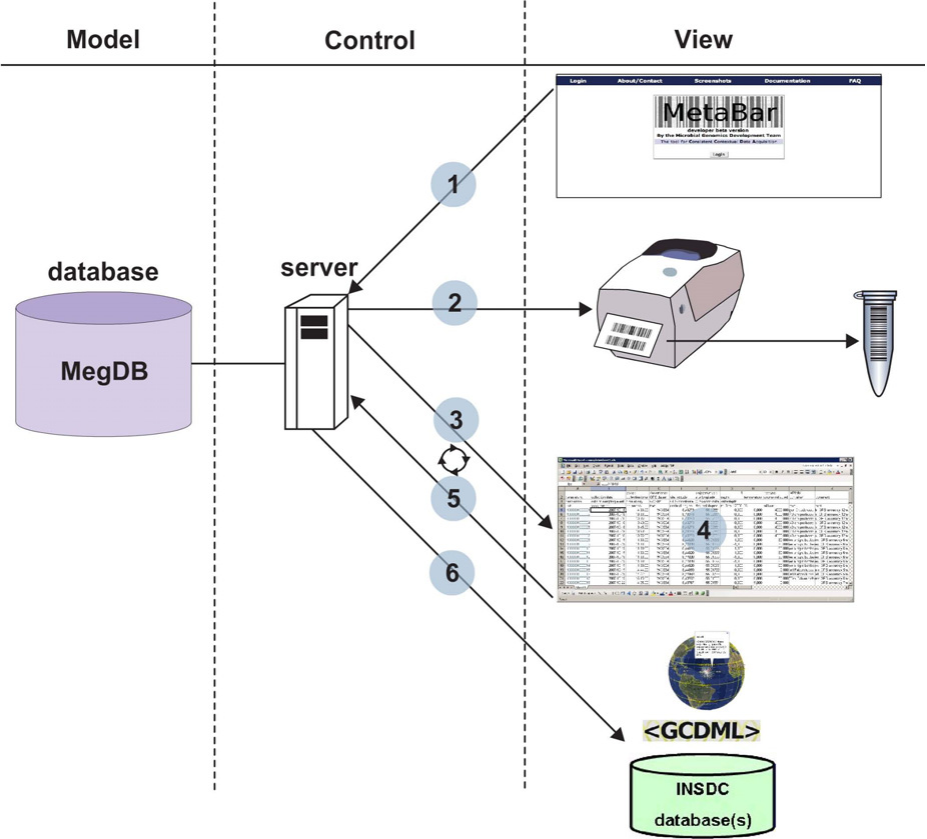
**Scheme of the MetaBar workflow**. Users are allowed to create barcodes, print them onto physical labels, and to capture, upload, and update contextual data for the barcodes using Excel^® ^spreadsheets. The contextual data can be exported to various formats and submitted to the INSDC databases.

**Figure 2:**
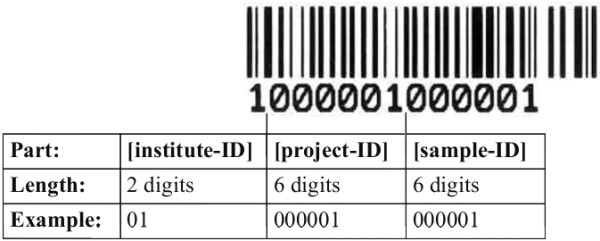
**The barcode identifier**. Barcodes consist of three parts ([institute-ID], [project-ID] and [sample-ID]) which in combination uniquely identify a sample. The barcodes are printed onto physical labels that can be placed on sample containers.

**Figure 3:**
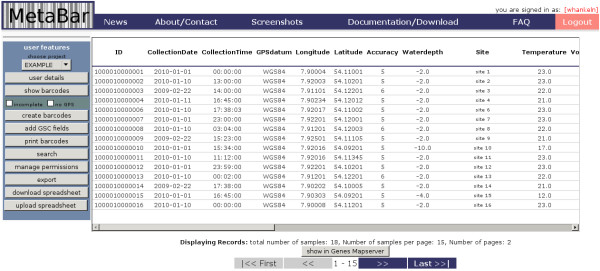
**Screenshot of the graphical user interface I**. Uploaded contextual data can be browsed and queried online.

The core application can be best explained by describing the workflow across the different MetaBar components (Figure 1). First, users log on to the MetaBar web server (Figure 1, step 1). Upon entry, users can allocate a certain range of sample identifiers before, during or after a sampling campaign. The identifier consists of a six digit [sample-id] that is incremented with every new sample, a six digit [project-id] that is incremented with every new project and a two digit [institute-id] that is fixed and identifies a certain institute. The combination of these three parts in one identifier assures the unique identification of each sample. The identifiers can be printed (Figure 1, step 2) as barcodes onto labels (Figure 2) that can be placed on sample containers and pasted into laboratory notebooks for consistency. Users can download (Figure 1, step 3) the acquisition spreadsheet containing the allocated identifiers and the empty contextual data fields in the first worksheet. As the user fills these fields, VBA validation macros check the inputs and users are prompted to use, for example, correct formats in the correct numerical range, where applicable. New worksheets can be added to the spreadsheet. Thus, any additional data outside of the MetaBar model can be added to the same file. Once the worksheets are filled (Figure 1, step 4) they can be uploaded (Figure 1, step 5) to the MetaBar web server. After the upload is finished, the file is parsed and the values in the first worksheet are stored in the respective relational fields of the central database. The additional worksheets in the file are not lost, but stored as binary data in the database. The latter three steps can be repeated whenever it is necessary to edit and update the data. Users can log in to the system at any time to search and browse their data via the web GUI (Figure 3).

**Figure 4:**
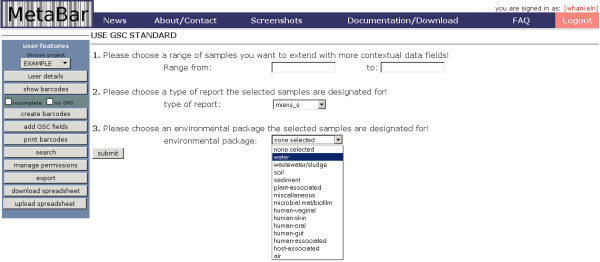
**Screenshot of the graphical user interface II**. Contextual data entries can be extended with GSC parameters. All contextual data can be exported and submitted to the INSDC databases.

Additionally, the MetaBar core set of contextual data fields can be extended for each sample with further GSC compliant parameters. These additional fields are organized into different types of report and environmental packages, each containing further parameters. The parameters can be directly selected and updated via the web interface (Figure 4).

MetaBar can also be used as an inventory e.g. for freezer contents. The database may be queried using sample identifiers by scanning their barcodes with an appropriate device, by manually entering their corresponding numeric code, or by text search on a metadata field. The query then retrieves all corresponding contextual data stored in the system.

MetaBar is integrated into a set of external tools with direct access from the web interface. The interpolation of physical and chemical parameters such as temperature, nitrate, phosphate, salinity, silicate, dissolved oxygen, oxygen saturation, apparent oxygen utilization and chlorophyll of a marine sampling site can be initiated via the WOA05 data extractor of the megx.net portal. On the fly visualization of sampling sites on a world map can be performed using the Genes Mapserver http://www.megx.net/gms. Furthermore, four export functions (Figure 1, step 6) are currently supported: (1) an export to KML to visualize sampling sites including their contextual data in Google Earth^®^, (2) an export to GCDML [19] for report creation and for data exchange, (3) an export to a GSC compliant MIGS/MIMS/MIENS spreadsheet, and (4) an export as a structured comment for sequence data submission to the INSDC databases using the Sequin tool http://www.ncbi.nlm.nih.gov/Sequin/index.html.

### Role, ownership and permission concept

MetaBar's user management provides a "MetaBar admin", a "project admin" and a "MetaBar user" mode. These modes depend on the role that is assigned to a certain user account. The web GUI possesses a cascading menu on the left which contains the "MetaBar admin features", the "project admin features", and the "MetaBar user features", respectively. Furthermore, a sophisticated ownership and permission concept offers the users to share their data with other users in the same project giving them read or write permissions, or to prevent access for others. Project admins have the possibility to transfer the ownership of a set of samples to another user, create projects, assign users to a project and to remove users from a project. For a given MetaBar installation there is only one MetaBar admin who can create users, assign or dismiss project admins and delete samples or whole projects.

A quick reference guide describing the general workflow of MetaBar from the acquisition to the submission of data is available on the website. Examples for metadata enriched INSDC database entries, created with MetaBar, are available through the accession numbers: [GenBank:GU949561 and GenBank:GU949562].

## Discussion

MetaBar can be used whenever it is necessary to capture contextual data that describe the environmental origin of a sample. The system has been tested in several studies in close collaboration with biologists taking samples in the field. By integrating their feedback MetaBar should qualify as user-friendly, scientist-centric software tool.

### Case study

The above mentioned studies allow the typical MetaBar workflow to be generalized as follows. A scientist acting as the project administrator (PA) plans a sampling campaign together with two members in his research team (PM1, PM2). The project EXAMPLE is created in MetaBar and the users PM1 and PM2 are added to EXAMPLE. It is anticipated that PM1 will collect five push core samples of sediment while PM2 will collect five water samples. Thus, PM1 and PM2 create five barcodes each and download their acquisition spreadsheets. These barcodes are printed in multiple copies to label sample containers, appear in field notebooks and for contingency.

During sample collection, PM1 and PM2 log contextual data such as latitude, longitude, depth and sampling time next to the barcode labels pasted in their field notebooks. The sample containers are labeled with the corresponding barcodes and transported back to the laboratory. The link between sample and environment is thus established. At the end of the sampling campaign the contextual data gathered in the field are transferred to PM1 and PM2's acquisition spreadsheets. Barcodes pasted on the sample, the field records and present in the spreadsheet ensure fidelity and the data are then uploaded to the MetaBar server over the internet. PM1 and PM2 may enter further contextual data specific to their sampling environments by selecting the relevant GSC-compliant metadata packages (e.g. "sediment" and "water", respectively) through the web GUI. The PA and both members of the project can now review the consolidated contextual data for errors or missing values. Corrective action at this stage improves the quality of the data prior to submission.

During laboratory processing, every new subsample is labeled with a copy of the original sample's barcode, preserving the link to the *in situ *sampling event. Native laboratory protocols and practices are otherwise unaffected and are documented in laboratory books. PM1 sequences the genomes of several sediment sample isolates and PM2 sequences microbial metagenomes from the community in the water sample. Congruent to the environmental extensions, GSC packages corresponding to various study types are available. PM1 and PM2 may use the "MIENS culture (miens_c)" and "metagenome (me)" packages, respectively, to record data specific to their study type (Figure 4). PM1 and PM2 receive their genome and metagenome sequences as FASTA files with automatically generated sequence identifiers in the header. The researchers enter these identifiers into the "seqID" field in the acquisition spreadsheet and export the data to a format for submission to INSDC. With this mapping, these contextual datasets can easily be combined with one or more FASTA sequences using a suitable submission tool. The researchers then submit their metadata-enriched sequences from the EXAMPLE project to an INSDC database.

MetaBar implements a neat trade-off between universality and specificity. The export functions assure that the collected data can be publicly stored and shared with the scientific community.

### Comparison of MetaBar and Handlebar

The idea of uniquely identifying samples and storing data about these samples in databases is not new and is widely used in many applications and disciplines. However, tools able to capture the contextual data of environmental samples combined with barcode labeling are rare. To our knowledge with the exception of MetaBar, the only open source tool using barcoding to identify georeferenced samples from the environment is Handlebar [20]. A tabular comparison of the programs' general features can be found in Table 1.

**Table 1:** Features of Handlebar and MetaBar

	Handlebar	MetaBar
Focus	Web-based lightweight LIMS for handling barcoded samples	Web-based tool for consistent contextual data acquisition with barcoded samples

System requirements	Operating system: Windows^® ^or GNU Linux Apache, Perl, PostgreSQL, OpenOffice or Microsoft^® ^Excel^®^	Without local MetaBar server installation: Operating system: Windows^® ^Internet connection, web browser (e.g. Firefox), Microsoft^® ^Excel^® ^2003 or higher Optional: EPL barcode printer (e.g. a Zebra^® ^TLP 2824) With local MetaBar server installation: Operating system: Windows^® ^or GNU Linux Apache, Java, Spring, jasig CAS, PostgreSQL, Microsoft^® ^Excel^® ^2003 or higher

Coverage	Metadata that emerges during sampling events and subsequent processing step data	Contextual data that emerges during sampling events (other data optional)

Sample type templates	Various	One generic and extensible template

Input validation	Done by the server	Done by VBA^® ^macros in the acquisition spreadsheet and on the server

Integration into data analysis tool set	GenQuery	http://www.megx.net

Export functions	-	GCDML, KML (for Google Earth)

Contextual data enriched sequence submission support	-	Export to MIGS/MIMS/MIENS and structured comment

HandleBar, as a lightweight LIMS, not only covers contextual data that are recorded during sampling, but also aims to document subsequent sample processing steps in the laboratory. In this respect, MetaBar is a simplification focusing only on the capture of contextual data in the field. MetaBar does not seek to replace well established laboratory bookkeeping or professional LIM systems, but rather aims to complement this process to ensure that contextual data are electronically accessible. Nevertheless, users may choose to use the tool as a storage inventory manager or to store intermediate results of sample processing because it is possible to store additional data in the spreadsheets. It is important to note that coupling contextual data with sequence data before submission to the INSDC databases is a unique feature of MetaBar.

The barcodes are, by concept, solely used to link environmental samples to contextual and, if available, sequence and species data derived from a labeled sample, thus, no hierarchy or processing method is encoded in the identifiers. Also, sample hierarchies and complex identifier schemes are avoided. This concept does not interfere with native laboratory sample tracking methods, yet ensures consistency in environmental contextual data capture.

It is important that users have the flexibility to cover different sample types. MetaBar offers a single template in which a restricted part is parsed to the database and an unrestricted part of the spreadsheet can be changed to contain sample specific additional data. HandleBar offers a set of non-constrained sample templates depending on the sample type and also individual templates can be created. In MetaBar each sample can be extended with further parameters organized into types of report and environmental packages suggested by the GSC.

In contrast to HandleBar, data entered into MetaBar's acquisition spreadsheet is validated on input, ensuring correct format before upload to the MetaBar server. This avoids frequent rejection of the acquisition sheet. In HandleBar the validation is done by the web server and erroneous sheets have to be corrected retrospectively by the uploading user. The variety of export features are currently unique to MetaBar.

MetaBar is integrated into the megx.net tool set and connected to MegDB. This offers opportunity to work with the data and to analyze them alone, or in the context of other research project data stored in the megx.net database. This level of integration necessitated a user authentication and authorization management system and SSL encryption. Consequently, the local installation of MetaBar requires modification of the open source code base. The software and a detailed installation manual are available at http://www.megx.net/metabar. However, accounts on the MetaBar installation hosted at the MPI for Marine Microbiology in Bremen can easily be given to interested users and an "anonymous" project exists where data of external users can be stored anonymously. It is the intention of the Microbial Genomics and Bioinformatics Group at the MPI-Bremen to support this tool as open source in the future.

### Applicability

MetaBar has been developed at the Max Planck Institute for Marine Microbiology; however, the tool may be readily applied to a wide range of research fields outside the marine sciences. Contextual data fields relevant to air, host associated, human associated, sediment, soil, wastewater sludge or water samples are available via the "add GSC fields" function. The parameters in each of these environmental packages have been selected based on community usage and consensus http://gensc.org/gc_wiki/index.php/MIENS. For example, fields requesting data on barometric pressure, carbon dioxide, carbon monoxide, chemical administration, humidity, methane, organism count, oxygen, oxygenation status of sample, perturbation, pollutants, respirable particulate matter, sample salinity, sample storage duration, sample storage location, sample storage temperature, solar irradiance, temperature, ventilation rate, ventilation type, volatile organic compounds, wind direction, and wind speed would be presented to users using the air environmental package. Users may easily add new, custom fields as columns using standard Microsoft^® ^Excel^® ^operations. Combined, the GSC extensions and freedom for customization generalize MetaBar's applicability to any scenario necessitating the capture of contextual data describing a sample's environmental origin.

## Conclusion

MetaBar offers an integrated contextual data acquisition, storage, and submission solution to the INSDC system. The impact of better contextual data availability and correctness in the primary sequence databases will greatly improve the possibilities to reach a higher level of data integration and interpretation to address basic ecological questions. MetaBar's integration into the megx.net tool set and its export mechanisms offer extended analysis possibilities via comparison to other scientific studies and with complementary interpolated environmental data. The visualization of the sampling sites on the Genes Mapserver and in Google Earth^® ^offers the users a simple way to show sampling events on the globe and to relate them to other publicly available scientific studies.

Statistical analysis of phylogenetic and functional biodiversity in their environmental context will reveal new insights into the biogeography and habitat adaptation of organisms. In the medical field, for example, it will be possible to create detailed disease maps which reveal mutation patterns of a certain pathogenic organism over time [5,11]. Such maps might help to predict the dispersal of epidemics and pandemics around the globe. For marine microbiology, Ed DeLong and coworkers have successfully shown that there is a stratification of genomic variability along the depth continuum in the water column at a specific sampling location [4]. It has also been demonstrated that specific diversity patterns are annually recurring [7]. A dense network of data points, enriched with contextual data, will lead to new insights into the complex interplay of organisms by comparing different sampling sites around the globe and over time. The denser this network of data points, the more will be revealed about the influence of the biotic factor in the elementary nutrient cycles that profoundly affect Earth's climate.

## Availability and Requirements

Project name: MetaBar

Software

Project homepage: http://www.megx.net/metabar

Operating systems: Linux and Windows

Programming language: Java JRE 1.5 or higher

Other requirements: Microsoft^® ^Excel^® ^2003 or higher, Google Earth^® ^(optional)

License: GNU General Public License version 3 (GNU GPL3)

Hardware

At least 1024 Mb of RAM

EPL barcode printer (e.g. a Zebra TLP 2824) (optional)

Barcode handscanner (optional)

The software can be tested anonymously using the login: "anonymous" with the password: "testmetabar".

## Authors' contributions

WH developed and implemented MetaBar and wrote the manuscript. RK advised programming design and helped with the integration of MetaBar with MegDB and megx.net. DF tested the software on cruises and provided feedback for design improvements. PY assured the MIGS/MIMS/MIENS standard compliance in MetaBar. PLB critically revised the manuscript and took care of EnvO integration. PY, PLB and RK tested the tool in the field. FOG supervised the work and helped with writing the manuscript. All authors read and approved the final manuscript.

## Supplementary Material

Additional file 1 **MetaBar open source code base under GNU GPL3 license**. Zipped open source code base for local installation. A general installation manual can be found in the README.txt. Click here for file
