# Supplementary figures and images for: MetaBar - a tool for consistent contextual data acquisition and standards compliant submission
Source: BMC Bioinformatics. 2010 Jun 30;11:358. doi: 10.1186/1471-2105-11-358 (PMC2912304; doi:10.1186/1471-2105-11-358)

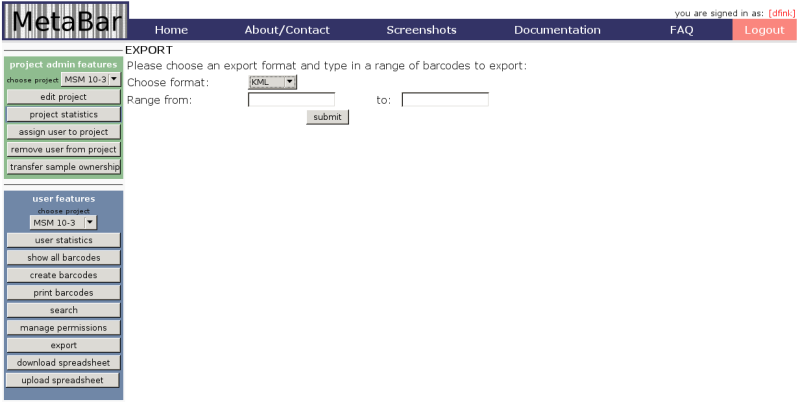

Supplement: Additional file 1 — MetaBar open source code base under GNU GPL3 license. Zipped open source code base for local installation. A general installation manual can be found in the README.txt. [file 1471-2105-11-358-S1.ZIP › metabar-1.1/war/img/metabar_6_medium.png]

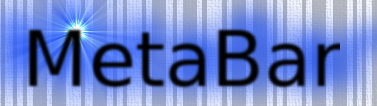

Supplement: Additional file 1 — MetaBar open source code base under GNU GPL3 license. Zipped open source code base for local installation. A general installation manual can be found in the README.txt. [file 1471-2105-11-358-S1.ZIP › metabar-1.1/war/img/metabar.png]

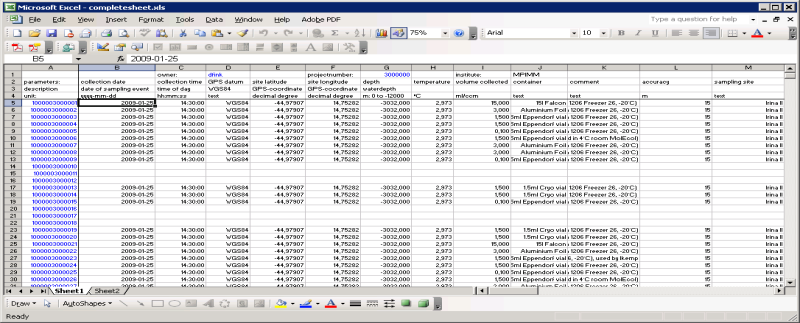

Supplement: Additional file 1 — MetaBar open source code base under GNU GPL3 license. Zipped open source code base for local installation. A general installation manual can be found in the README.txt. [file 1471-2105-11-358-S1.ZIP › metabar-1.1/war/img/metabar_1_medium.png]

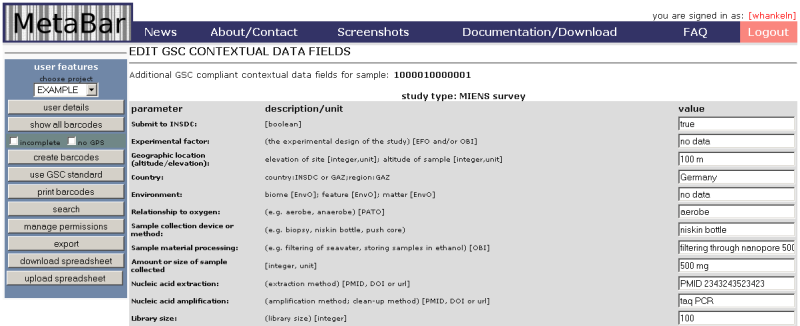

Supplement: Additional file 1 — MetaBar open source code base under GNU GPL3 license. Zipped open source code base for local installation. A general installation manual can be found in the README.txt. [file 1471-2105-11-358-S1.ZIP › metabar-1.1/war/img/FIGURE5_medium.png]

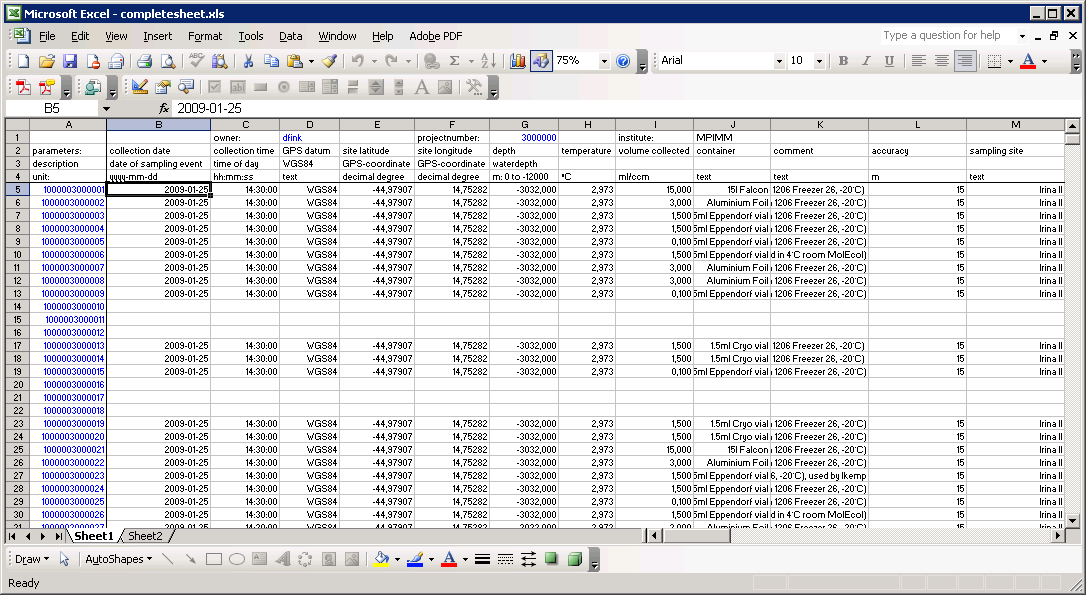

Supplement: Additional file 1 — MetaBar open source code base under GNU GPL3 license. Zipped open source code base for local installation. A general installation manual can be found in the README.txt. [file 1471-2105-11-358-S1.ZIP › metabar-1.1/war/img/metabar_1.png]

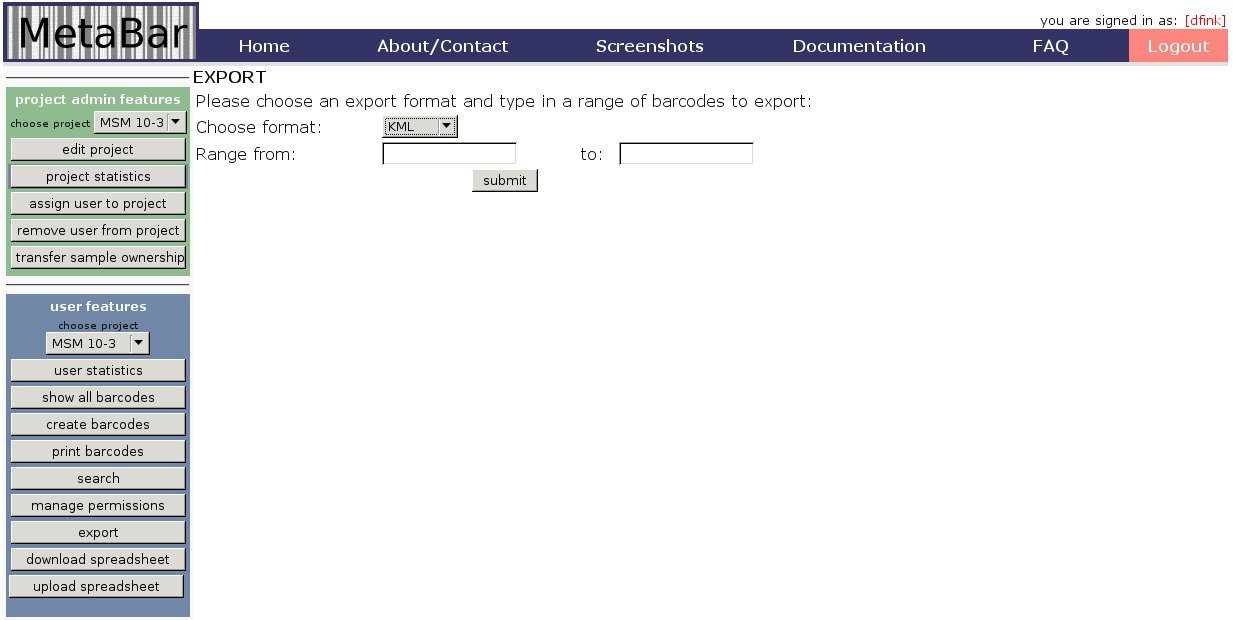

Supplement: Additional file 1 — MetaBar open source code base under GNU GPL3 license. Zipped open source code base for local installation. A general installation manual can be found in the README.txt. [file 1471-2105-11-358-S1.ZIP › metabar-1.1/war/img/metabar_6.png]

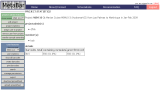

Supplement: Additional file 1 — MetaBar open source code base under GNU GPL3 license. Zipped open source code base for local installation. A general installation manual can be found in the README.txt. [file 1471-2105-11-358-S1.ZIP › metabar-1.1/war/img/metabar_3_small.png]

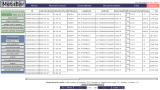

Supplement: Additional file 1 — MetaBar open source code base under GNU GPL3 license. Zipped open source code base for local installation. A general installation manual can be found in the README.txt. [file 1471-2105-11-358-S1.ZIP › metabar-1.1/war/img/metabar_2_small.png]

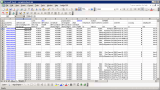

Supplement: Additional file 1 — MetaBar open source code base under GNU GPL3 license. Zipped open source code base for local installation. A general installation manual can be found in the README.txt. [file 1471-2105-11-358-S1.ZIP › metabar-1.1/war/img/metabar_1_small.png]

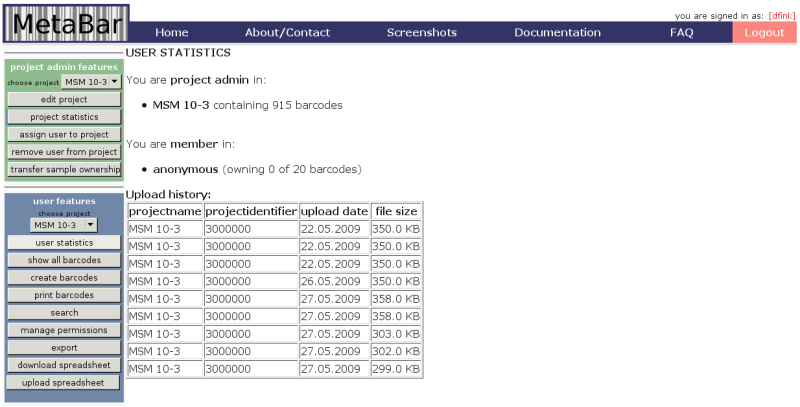

Supplement: Additional file 1 — MetaBar open source code base under GNU GPL3 license. Zipped open source code base for local installation. A general installation manual can be found in the README.txt. [file 1471-2105-11-358-S1.ZIP › metabar-1.1/war/img/metabar_4_medium.png]

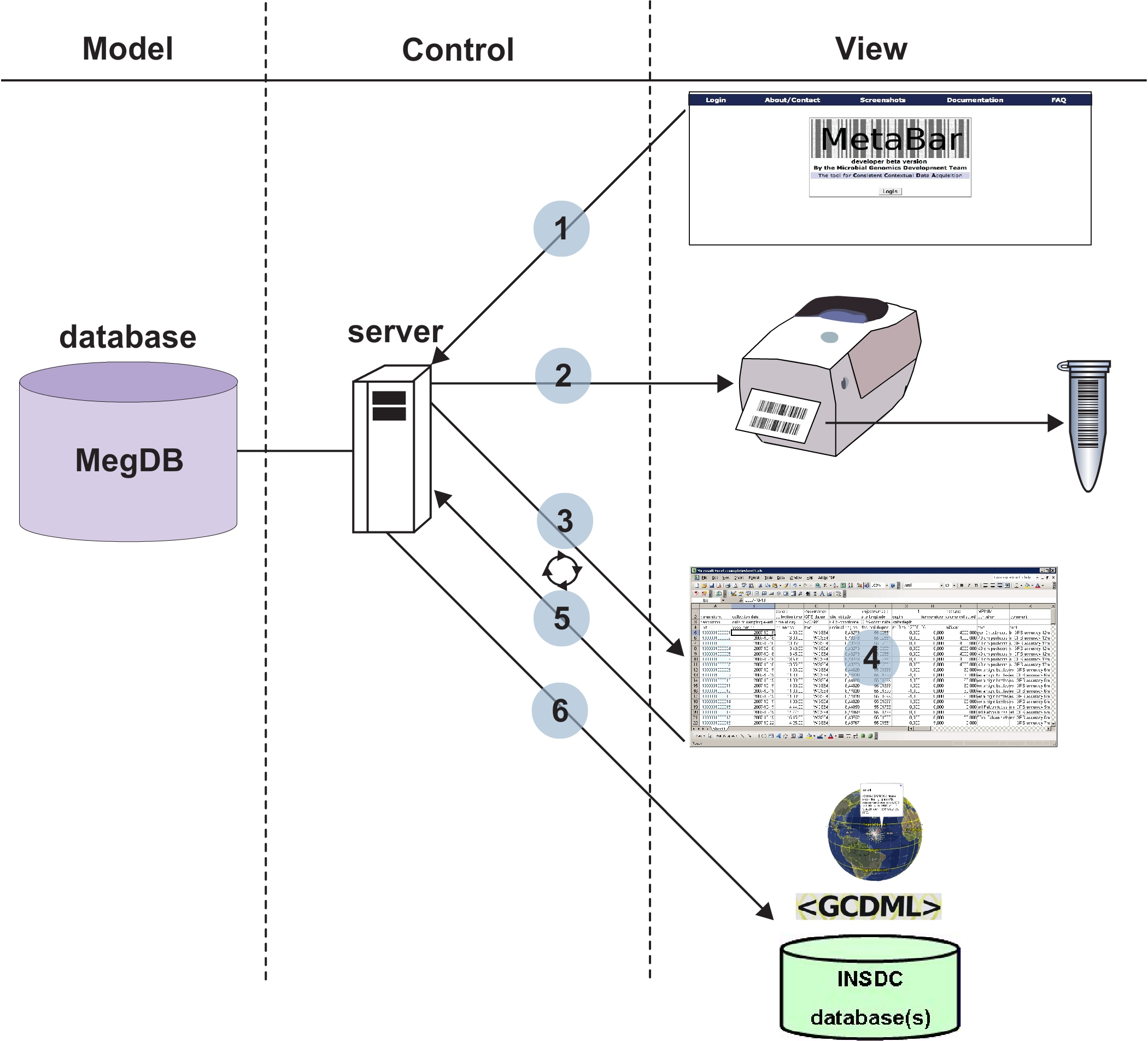

Supplement: Additional file 1 — MetaBar open source code base under GNU GPL3 license. Zipped open source code base for local installation. A general installation manual can be found in the README.txt. [file 1471-2105-11-358-S1.ZIP › metabar-1.1/war/img/metabar_workflow_complete.png]

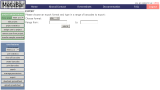

Supplement: Additional file 1 — MetaBar open source code base under GNU GPL3 license. Zipped open source code base for local installation. A general installation manual can be found in the README.txt. [file 1471-2105-11-358-S1.ZIP › metabar-1.1/war/img/metabar_6_small.png]

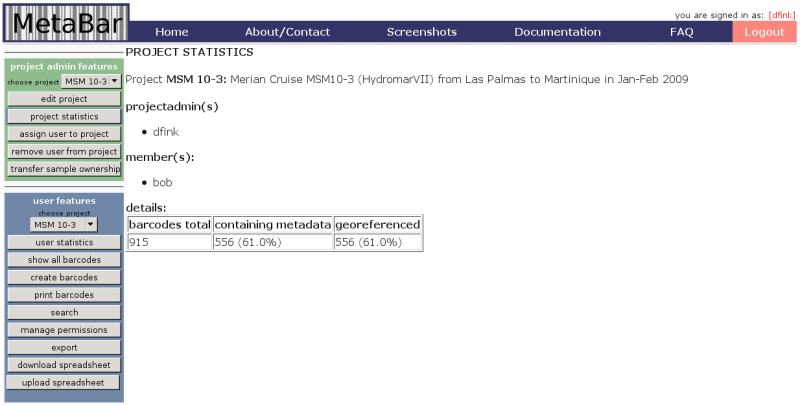

Supplement: Additional file 1 — MetaBar open source code base under GNU GPL3 license. Zipped open source code base for local installation. A general installation manual can be found in the README.txt. [file 1471-2105-11-358-S1.ZIP › metabar-1.1/war/img/metabar_3_medium.png]

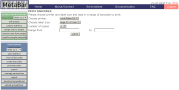

Supplement: Additional file 1 — MetaBar open source code base under GNU GPL3 license. Zipped open source code base for local installation. A general installation manual can be found in the README.txt. [file 1471-2105-11-358-S1.ZIP › metabar-1.1/war/img/metabar_5_small.png]

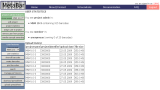

Supplement: Additional file 1 — MetaBar open source code base under GNU GPL3 license. Zipped open source code base for local installation. A general installation manual can be found in the README.txt. [file 1471-2105-11-358-S1.ZIP › metabar-1.1/war/img/metabar_4_small.png]

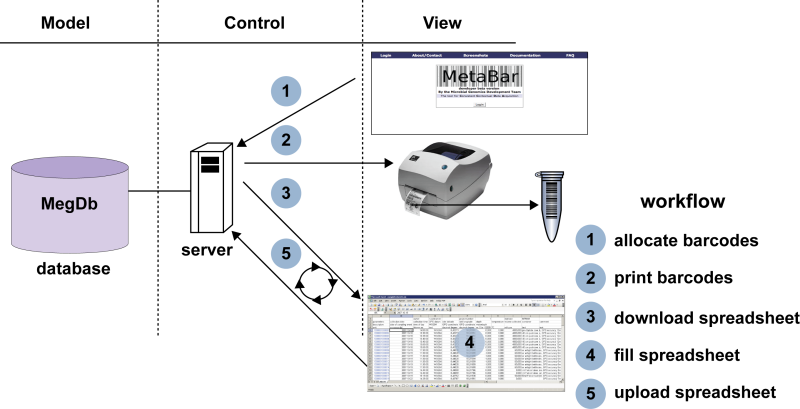

Supplement: Additional file 1 — MetaBar open source code base under GNU GPL3 license. Zipped open source code base for local installation. A general installation manual can be found in the README.txt. [file 1471-2105-11-358-S1.ZIP › metabar-1.1/war/img/metabar_workflow_medium.png]

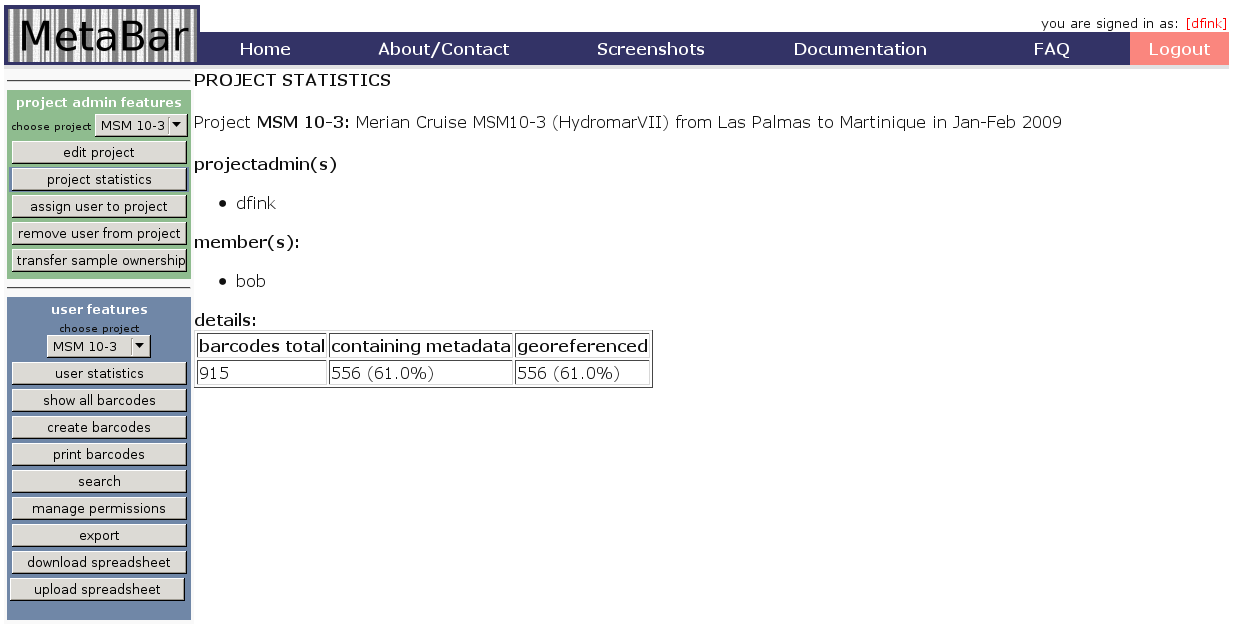

Supplement: Additional file 1 — MetaBar open source code base under GNU GPL3 license. Zipped open source code base for local installation. A general installation manual can be found in the README.txt. [file 1471-2105-11-358-S1.ZIP › metabar-1.1/war/img/metabar_3.png]

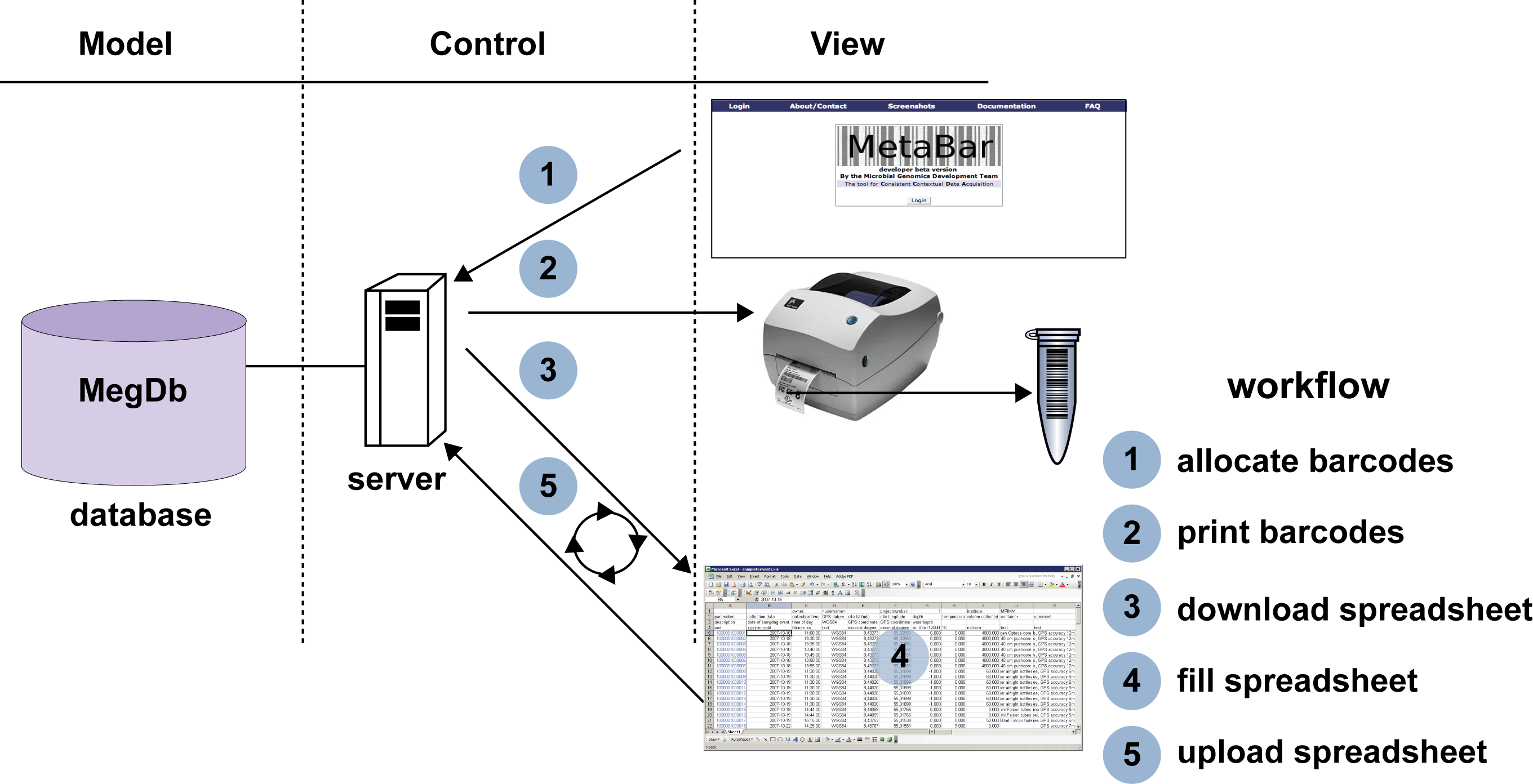

Supplement: Additional file 1 — MetaBar open source code base under GNU GPL3 license. Zipped open source code base for local installation. A general installation manual can be found in the README.txt. [file 1471-2105-11-358-S1.ZIP › metabar-1.1/war/img/metabar_workflow.png]
